# Organic Charge Transfer Cocrystals as Additives for Dissipation of Contact Charges on Polymers

**DOI:** 10.1021/acsami.2c13643

**Published:** 2022-12-06

**Authors:** Sunay Dilara Ekim, Görkem Eylül Kaya, Murat Daştemir, Erol Yildirim, H. Tarik Baytekin, Bilge Baytekin

**Affiliations:** † UNAM National Nanotechnology Research Center, Bilkent University, Ankara 06800, Turkey; § Department of Chemistry, Middle East Technical University, Ankara 06800, Turkey; ⊥ Polymer Science and Technology Program, Middle East Technical University, Ankara 06800, Turkey; || Department of Chemistry, Bilkent University, Ankara 06800, Turkey

**Keywords:** charge transfer complexes, antistatic, contact electrification, polydimethylsiloxane, composites, polymers

## Abstract

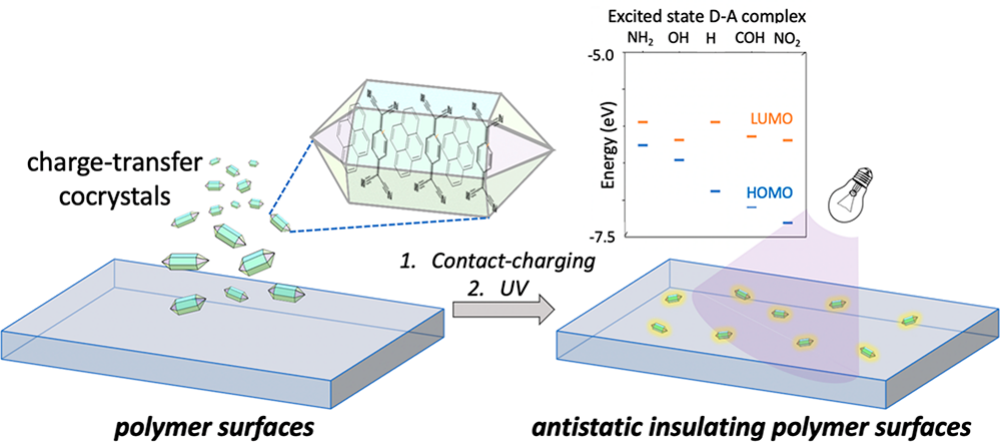

Common polymers can accumulate surface charges through contact, a phenomenon known since ancient times. This charge accumulation can have detrimental consequences in industry. It causes accidents and yields enormous economic losses. Many empirical methods have been developed to prevent the problems caused by charge accumulation. However, a general chemical approach is still missing in the literature since the charge accumulation and discharging mechanisms have not been completely clarified. The current practice to achieve charge mitigation is to increase materials conductivity by high doping of conductive additives. A recent study showed that using photoexcitation of some organic dyes, charge decay can be started remotely, and the minute amount of additive does not change the material’s conductivity. Here, we show the contact charging and charge decay behavior of polydimethylsiloxane doped with a series of organic charge transfer cocrystals (CTC) of TCNQ acceptor and substituted pyrene donors (CTC-PDMS). The results show that the CTC-PDMS are antistatic, and the discharging propensity of the composites follows the calculated charge transfer degree of the complexes. On the other hand, the CTC-PDMS are still insulators, as shown by their high surface resistivities. Kelvin probe force microscopy images of the contact-charged and discharged samples show a quick potential decay in CTC domains upon illumination. Combined with the fast overall decay observed, the antistatic behavior in these insulators can be attributed to an electron transfer between the mechanoions in the polymer and the CTC frontier orbitals. We believe our results will help with the general understanding of the molecular mechanism of contact charging and discharging and help develop insulator antistatics.

The contact of two insulator polymers creates charges on the polymer surfaces. This simple phenomenon, contact charging^1−4^ (aka static charging, tribocharging), leads to a large (kilovolts) electric potential on the surfaces. The static charges can remain on the surfaces of isolated polymers for weeks. Indeed, this “charge stability” causes million-dollar problems in electronics, aviation, space, pharmaceuticals, textiles, and polymer manufacturing.^5−7^ Contact charging is also a significant contributor to friction and wear in devices made up of insulators.^8,9^ Since synthetic and natural polymers can get contact charged, the problem exists for all insulating polymers.^10^ Therefore, it is necessary to address the chemical charge stability and charge dissipation to solve the issues related to the static charging of common polymers. However, this is not an easy task since the molecular-level mechanism for charge formation on polymers is not clear yet.^11^ This is not surprising since the contact charging of polymers includes complex events, including electron and ion transfer, bond breaking,^12−15^ and material transfer^16−21^ between the surfaces. All these events depend strongly on the chemical, physical (e.g., roughness, hydrophobicity), mechanical properties (e.g., elastic modulus, hardness) of the polymers, and environmental conditions (e.g., humidity, temperature). Also, the charges can form during both contact and separation of the polymer surfaces.^22^

Contact charges on the polymers can be discharged by several methods. The commercialized conventional methods involve doping the polymers with additives^23^ to increase the surface conductivity.^24^ This can be achieved by adding conducting additives, e.g., metals and metal nanoparticles, or increasing the amount of water adsorbed on the polymer surfaces by adding salts.^25^ The latter can also be realized by the surface oxidation of the polymers.^26^ Considering the electron transfer mechanism, the surface modifications can also be used to render polymers antistatic.^27^ In the past decade, we^28^ and others^29,30^ have also shown an unconventional charge dissipation mechanism involving organic radical scavengers (e.g., tocopherol, diphenylpicryl hydrazyl, dopamine, and tannic acid) doped into the polymers. These studies demonstrated that rendering polymers antistatic without altering their conductivity is possible. This discovery provided a way to make polymers antistatic without changing the other electrical, mechanical, and optical properties of the polymer materials. Later, it was found that this radical scavenging mechanism is the basis of the antistatic behavior of wood, an insulator material.^31^

In a previous study, we used light to dissipate charges on common polymers doped with organic dyes to control charge dissipation remotely.^31^ In that report, it was stated that the higher the dipole moment of the excited dyes, the faster the charge dissipation. Building on this knowledge, here, we explore organic charge transfer cocrystals (CTC) as antistatic additives for contact charge mitigation on polymers. CTCs have inherent dipole moments, which can be enhanced by illumination, making them exciting candidates as such additives.

CTCs^33−35^ are ordered assemblies of multiple (usually two) components, a donor and an acceptor stacked either segregated or mixed. The assembly is through secondary interactions and is facilitated further by inherent charge transfer between the components.^36^ The CTCs can have many interesting properties; they can be insulators or conductive like a metal. They can display ferroelectricity, and nonlinear optical properties, which resulted in their widespread use in stimuli-responsive materials.^37−39^ Different crystal packing modes of CTCs add to the observed property versatility. The preparation of CTCs is straightforward and involves the physical mixing of the two organic counterparts in solution or the solid-state.

As mentioned above, it is possible to discharge contact-charged common polymers with photoexcitation of the polar organic dye additives.^32^ CTCs, too, are polar due to the charge separation (ρ) in the ground state, which gets higher when they are promoted to the excited state upon illumination. Additionally, in CTCs, this polarity can be controlled by affecting the charge separation by choosing donor and acceptor molecules with appropriate energy levels. In this way, the bandgap values can also be tuned. This report shows that when ambipolar insulator CTCs are added to a polymer matrix, they can act as antistatic agents and help the dissipation of charges on contact-charged polymers. We report illumination and the magnitude of donor–acceptor (D–A) charge separation can affect the contact-charge dissipation rates on polymers doped with CTCs.

To test our hypothesis that CTCs can behave as antistatic additives, we prepared solid polymers doped with CTCs formed from a set of donors and an acceptor. We chose polydimethylsiloxane, PDMS (Sylgard 184), as the polymer matrix, a benchmark material for polymer contact-charging research.^40^ PDMS prepolymer can be cured flat on surfaces, preventing errors from the different roughness samples. In our experiments, we doped such flat-surface PDMS samples with the CTC formed in solutions (1.0 × 10^–2^ M, CH_2_Cl_2_, Figure S1, for the UV–vis spectra of the donors, CTCs in solution and the solid state, Figure S2 for the XRD of the CTC-doped PDMS). There are numerous options for organic donors and acceptors forming CTCs. For the initial experiments, we selected a series of donors, pyrene and its derivatives, and tetracyanoquinodimethane, TCNQ, as our acceptor (Figure 1a). TCNQ^41^ is the most widely studied acceptor in CTCs.^33^ We preferred to start with unsubstituted pyrene since 1) in our previous study,^32^ pyrene affected no discharging (also under illumination) of the contact-charged polymer; 2) the pyrene-TCNQ pair forms a CTC, which is not conductive (∼10^–12^ S cm),^42^ considered as nonionic at room temperature, and has a low degree of charge separation^43^). The PDMS samples (discs with a radius of 0.90 cm and thickness of 1.52 mm, see the Supporting Information for preparation) were then immersed in vials of donor–acceptor solution in dichloromethane (30 mL, 1:1 mol ratio, each compound 1.0 × 10^–2^ M). In polar solutions, pyrene and TCNQ form a 1:1 complex with a room temperature equilibrium constant of ∼10.^44^ These complexes let the formation and deposition of micron-sized CTCs on the PDMS surface (Figure 1a). After drying, the samples displayed a band at 488 and 777 nm for 1:1 pyrene-TCNQ in PDMS in the UV–vis spectrum showing the charge transfer in the cocrystals embedded in the polymer matrix (similar to the ones reported in the literature for these complexes and their CTCs in the solid state^44^).

**Figure 1:**
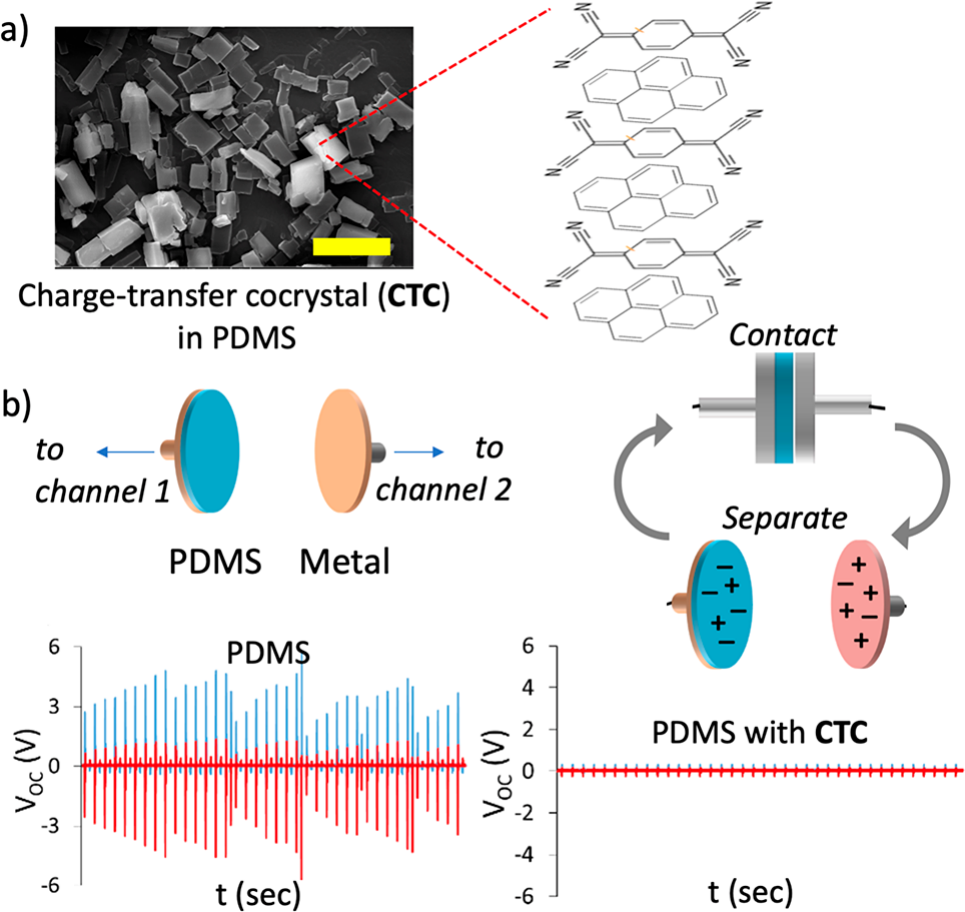
Pyrene–TCNQ charge transfer cocrystal (CTC) doped in PDMS can prevent the accumulation of contact charges on PDMS. (a) Representative SEM image of PDMS discs used in the tapping experiments showing micrometer-scale CTCs in the matrix. Scale bar: 5.0 μm. (b) Tapping setup for monitoring the polymer charging. Open circuit (*V*_oc_) electrical signals obtained from an aluminum electrode (red) that is tapped to a PDMS disc with a 5 Hz frequency (radius = 0.90 cm, thickness 1.52 mm, doping solution = 1.0 × 10^–2^ M) and from the metal electrode placed behind the PDMS surface (blue). The difference in the acquired contact charges between the CTC-doped and undoped PDMS can be visualized by up to a 10-fold decrease in the generated *V*_oc_ (here, data shown for undoped PDMS (left) and 1-aminopyrene-TCNQ doped PDMS (right)). See the Experimental Section for further details on the sample preparation and the tapping setup.

We used the two common literature methods to determine the contact charging (and the charge decay) on the doped and undoped PDMS pieces. In the first one, we used a tapping device on which the polymer pieces can be mounted on one of the electrodes, and on the other electrode is a metal (Al in our case). The electric potential developed on each electrode (*V*_oc_) upon contact and separation can be measured and recorded independently by a two-channel oscilloscope (see the Experimental Section for the details of the setup and the measurement of the *V*_oc_). The first results of these tapping experiments showed that the CTC-doped PDMS decreased *V*_oc_ to less than 10% of the values obtained by undoped PDMS (shown in Figure 1b, 1-aminopyrene/TCNQ).^45^

In the second setup, the pieces were manipulated by tweezers and charged against Al metal by consecutive touches until they reached a −2.5 nC surface charge, measured by a homemade Faraday cup connected to an electrometer (Keithley 6517B) (Figure 2a). The pieces were then exposed to the air in the Faraday cup and their discharge was recorded. The experimental discharge rates can be obtained from the charge vs time plots (Figure 2b, yellow solid line), which fit first-order decay for the first 30 min of discharge. (A full discharge may take hours to complete for undoped PDMS. We report only the initial discharge kinetics of the samples here.) The discharge data shows that the contact charges on CTC-doped PDMS decay faster (e.g., pyrene-TCNQ PDMS has 0.006 ± 0.001 s^–1^) than those on an undoped PDMS or a pyrene-doped PDMS (0.002 ± 0.001 s^–1^) when all pieces are contact charged to the same initial charge (Figure 2b, blue solid line). On the other hand, TCNQ can cause a distinctly faster discharge (0.061 ± 0.022 s^–1^) of charged PDMS when it is doped into the polymer on its own. This rapid discharge can be attributed to the electron transfer between the mechanoanions (forming during contact/separation) and the TCNQ.^13^ Under illumination with a UV-lamp that matches the CTCs’ absorption profiles (see Figure S1d), the CTC-doped PDMS discharge is further enhanced (Figure 2b, yellow dashed line) (0.014 ± 0.006 s^–1^ for pyrene/TCNQ cocrystal). These results show that CTC doping facilitates PDMS charge decay. (We note here that TCNQ-enhanced discharge is not accelerated by illumination.)

**Figure 2:**
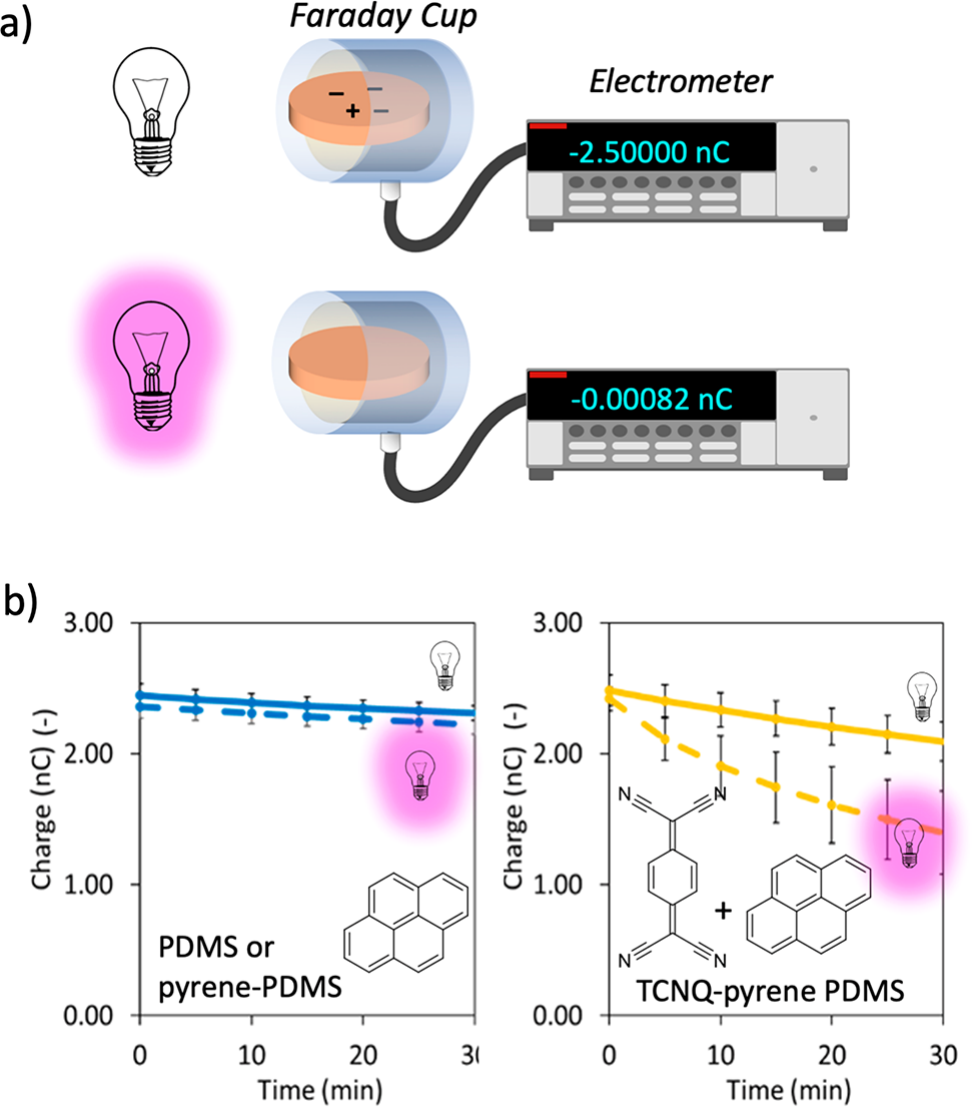
Contact-charging setup for monitoring the polymer charging. (a) Doped and undoped PDMS pieces are contact charged. The charges are let to decay (with and without UV illumination) in a Faraday cup connected to an electrometer recording charges. (b) Recorded charge decay is faster in the CTC-doped PDMS than that on pyrene-doped PDMS and undoped one (the latter two show identical decay behavior). The decay rate is further enhanced under illumination for CTC-doped PDMS, providing implications for CTC-charge interactions. Error bars in b correspond to standard deviations determined from at least four independent experiments. See Experimental Section for further details on sample preparation, discharge measurement setup, and Table S1 for rates calculated from charge decay experiments.

One can expect that CTC-mediated charge dissipation can be controlled by the degree of inherent charge transfer between the donor and acceptor molecules in the CTCs. For this purpose, we formed 1:1 dichloromethane solutions of the acceptor TCNQ with different pyrene donors; 1-nitropyrene, 1-pyrenecarboxaldehyde, 1-aminopyrene, and 1-hydroxypyrene. The acceptor and donors form soluble assemblies in dichloromethane. In a series of experiments, we used 35 polytetrafluoroethylene (PTFE, 1.6 mm diameter) beads contact charged by shaking in 8.0 mL hexane in glass vials using a vortexer. After 30 s of shaking, the beads acquired enough contact charges (−170 ± 55 pC) and adhered to the walls of the vials by electrostatic attraction. Hexane has a low dielectric constant (similar to air) that does not interfere with contact charging and provides the medium for the organic reagents to dissolve homogeneously.^32^ Then, dichloromethane solutions of the assemblies (2.5 μL, 1.0 × 10^–2^ M) were added to these glass vials with contact-charged beads. This setup, shown in Figure 3a, provides better statistics about discharging propensities than the solid polymer doping experiments shown above in Figure 2. In hexane only or a solution of pyrene derivatives, PTFE beads preserve their charges and hang on the inner walls of the glass vial for many hours to days. However, PTFE beads discharged and fell to the bottom of the vial in the first few minutes after adding charge transfer (CT) assembly solutions into the vial (Figure 3a and Movies S1 and S2). We monitored the discharge effectiveness of the various CT assembly solutions with the number of beads discharged in the first 500 s of discharge. Figure 3b, blue bars show the number of beads discharging in the CT assembly solutions increasing in the order of TCNQ assemblies of 1-nitropyrene < 1-pyrenecarboxaldehyde < pyrene < 1-aminopyrene ≤ 1-hydroxypyrene; these numbers increased further under illumination with UV (Figure 3b, orange bars).

**Figure 3:**
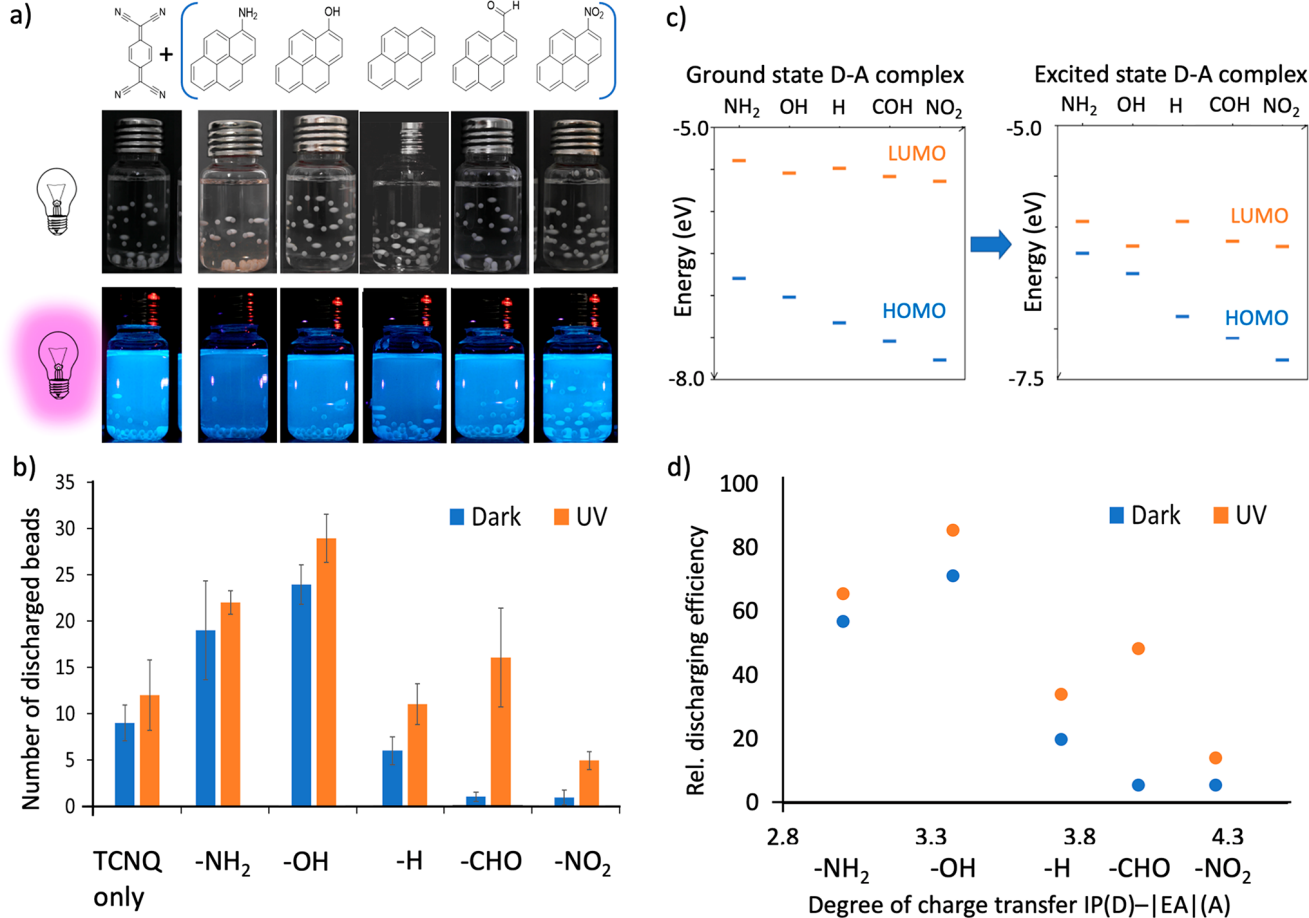
CT assembly mediated discharging of contact-charged polymer beads in hexane. (a) 35 PTFE beads in 8.0 mL of hexane are contact charged by shaking on a vortexer for 30 s. The charged beads “stick” electrostatically to the walls of the vials. Then CT assembly solutions of pyrene and its derivatives (donors) and TCNQ (acceptor) (1:1, final concentration 3.12 × 10^–6^ M, dry hexane) are added to glass vials. (b) In vials with CT assembly solutions, the beads discharge in ca. 500 s and fall to the bottom of the vial. UV illumination speeds up the process. There is no bead discharge for days in hexane only or just with the donor solutions of the same concentrations. Error bars correspond to standard deviations determined from five independent experiments. (c) Propensities toward faster charge decay in (b) follow the trends for closer HOMO–LUMO gaps (left), which are further narrowed by light (right). (d) Affected bead discharge, calculated from the ratio of the number of beads discharge to the total number of beads, in comparison to the degree of D–A charge transfer. The degrees of CT for the CTCs are calculated as IP(D)-EA(A) in the assemblies, lower values show a larger CT extend. For details, see the Experimental Section and Movies S1 and S2. For the details of the HOMO–LUMO and the charge separation calculations in CT assemblies, see the Supporting Information.

To check whether this discharge order aligns with the order for the charge transfer degree of the assemblies, we calculated the HOMO and LUMO energy levels of CTCs with DFT methods, Figure 3c (see the Supporting Information and Figure S3 for details of calculation). The calculations suggest that the gap between the HOMO and the LUMO energy levels decreases in the order 1-nitropyrene, 1-pyrenecarboxaldehyde, pyrene, 1-hydroxypyrene, 1-aminopyrene. Therefore, in this order, one can assume an increasing D–A charge transfer in the donor–acceptor assemblies. The calculations of the charge transfer value (for pairwise and quadruple CT assemblies, Table S3) by ground state electrostatic potential (ESP) and natural population analysis (NPA) verify this increase in the extent of D–A charge transfer. The IP(donor) – |EA|(acceptor) offset values (Table S3, lower values show a larger CT extent) and the bond length changes in the TCNQ molecules upon formation of the assemblies (Table S5, Figure S6) suggest the same charge transfer trend that is indicated by the HOMO–LUMO gap values. We then compared the calculated CT values to the success of the discharging of beads in solution, which is taken as the ratio of the number of discharged beads to the total number of beads (Figure 3d). It can be concluded that the degree of charge transfer of the CT assemblies in the solution parallels the success of discharging mediated by the assemblies. Furthermore, the increase in the bead discharge rate upon illumination is in line with a general decrease in the band gap of the photoexcited assemblies. (Figure 3c). Here, we note that the increase in the discharging efficiency upon illumination is related to the distance of the light source from the sample, the intensity of the source, and the proper choice of the source emission wavelength (matching the absorption profile of the CT assembly), which we kept constant during the experiments for all derivatives. In addition to these parameters, the choice of substitution on the donor affects the discharge rate enhancement upon illumination. The individual differences between the discharging efficiencies in dark and under illumination is least pronounced for the CT assemblies with the highest and lowest degrees of D–A charge transfer.

Encouraged by the data of the solution experiments and the observed discharging trend following the amount of charge transfer, we wondered whether similar trends could be observed in the solid-state polymer-CTC composites. Using identical preparation for the pyrene-TCNQ doped samples shown in Figure 2, we doped solid PDMS with the other CTCs we had. In all cases, the CTC in the polymer causes a rapid discharge of the contact charges, and further illumination facilitates the discharge rate. The degree of CT vs discharge rate relation holds in the solid state, too. The difference between the dark and UV-illuminated discharge was found to be lowest in both extremes of the charge transfer series. 1-Aminopyrene-TCNQ/PDMS discharges in the dark (solid line) and under UV illumination (dashed line) with similar rates, as shown in Figure 4.

**Figure 4:**
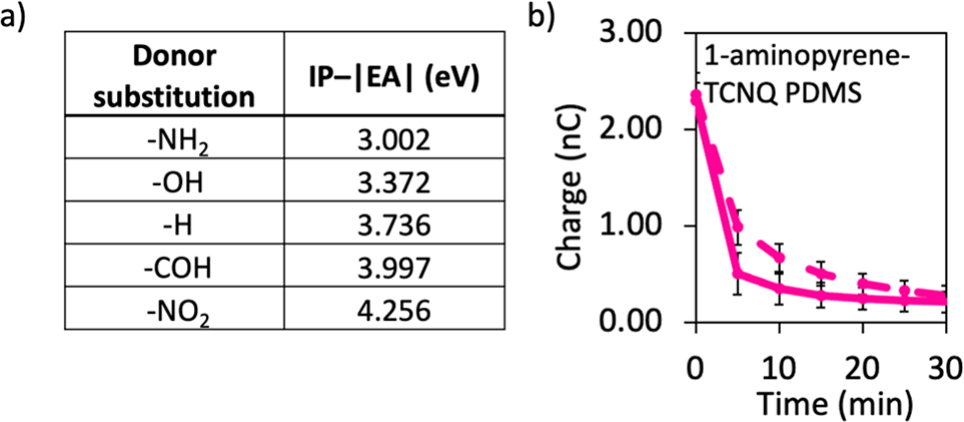
(a) Degree of the charge transfer calculated as IP(donor) – |EA|(acceptor) in the CT assemblies of the donor substituted pyrenes and the acceptor TCNQ. (b) Doped with the highest CT D–A pair, 1-aminopyrene-TCNQ, PDMS discharges in the dark (solid line) and under UV illumination (dashed line) with similar rates (compare this profile with the ones in Figure 2). RH = 40–55%. Error bars correspond to standard deviations determined from four independent experiments.

The discharge observed in solution and solid state may suggest that the discharge is independent of the concentration or morphology of the assemblies (in solution) or crystals (in the solid-state). We showed that this was not the case. Higher concentrations of the CT assemblies having needle-like morphologies that provide interconnectivity of the crystals contribute to the successful fast discharge, as shown in Figure 5. Finally, a more refined look at Figure 3d, showing a rough trend, suggests that although the degree of CT vs discharge rate relation can be used as an initial guide, other physical parameters related to the CT assemblies could also affect and contribute to this rough trend.

**Figure 5:**
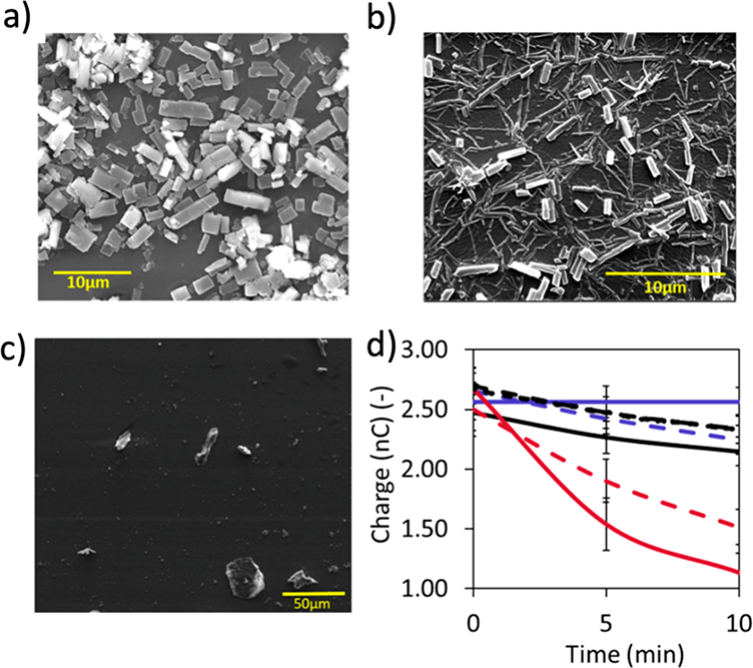
CTC morphology and CTC-mediated discharge. SEM images of PDMS doped with (a) pyrene-TCNQ and (b) 1-aminopyrene-TCNQ (doped in 1.0 × 10^–2^ M 1:1 CT assembly solutions in dichloromethane). Interconnectivity through needle-like CTC morphology in (b) may contribute to faster dissipation in this CTC (Figure 4). (c) Reducing the concentration of 1-aminopyrene-TCNQ doped PDMS to 1.0 × 10^–3^ M disrupts the interconnectivity of the crystals and reduces the charge dissipation effect of the CTCs, as shown in (d) the comparison of charge vs time plots; red lines for 1.0 × 10^–2^ M vs purple lines for 1.0 × 10^–3^ M (solid line = dark, dashed line = under illumination). The discharge experiments are obtained in 20–40% RH. Error bars correspond to standard deviations determined from four independent experiments.

We showed above that the faster discharge of contact charges on polymers is related to the presence of the CT assemblies and the degree of charge transfer in the assemblies. Next, we wondered about the possible chemical mechanism causing this fast charge dissipation. In common polymers, the charge dissipation of contact charges is traditionally facilitated by increased conductivity. As we mentioned previously, this increase is achieved by adding metal and metal nanoparticles or humidity-enhancers. In our CTC polymers (with CTC doping from 1.0 × 10^–2^ solutions), the measured surface conductivities are, e.g., 8.86 × 10^–14^ S for pyrene-TCNQ/PDMS, 8.33 × 10^–14^ S for 1-aminopyrene-TCNQ/PDMS. These values show that the composite material is an insulator. The illumination we used to facilitate the discharging does not affect these surface conductivity values (8.82 × 10^–14^ S for pyrene-TCNQ/PDMS, 8.33 × 10^–14^ S for 1-aminopyrene-TCNQ/PDMS). These values show that the charge dissipation does not originate from an overall surface conductivity increase.

If it is not the increase in the surface conductivity, what is the mechanism for the charge dissipation mediated by the presence of CTCs in the polymers? The exact mechanism of the contact-charge dissipation in common insulator polymers is hard to determine because of the inherent insulator nature of the polymers and the lack of proper instrumentation to identify the charge carriers.^11^ One possible explanation can be a mechanism that starts with the formation of the stable mechanoions (the broken bonds with ionic ends, which are indeed the “contact charges”) that form through the bond-breakages during the mechanical contact and separation.^13,18,28,46^ It was shown that the formed mechanoions can interact with the electron acceptors and transfer electrons to them.^13,47^ We surmise that after the formation of the mechanospecies on the contact-charged polymers doped with CTCs, electrons are transferred from the mechanoanions to the CTCs, which mediate a faster discharge, and the negative net charge on the polymers is reduced. To test this idea, we performed Kelvin probe force microscopy (KPFM) analyses on the CTC-doped PDMS samples before and after contact charging, and also upon a subsequent illumination. As displayed in Figure 6, before contact charging, the KPFM surface potential maps of the 1-aminopyrene-TCNQ/PDMS surfaces showed the regions of positive and negative charges on both the PDMS (as expected from the previous studies^4^) and CT crystal domains. Although the electrical potential probed with KFM on the domains is nonzero, the samples had no net charge (the net surface charge measured in the Faraday cup <50 pC). Upon contact charging with Al foil, the surface potential on the crystals shifts to more positive values (above +5.0 V), and PDMS gained negative charges (up to −1.0 V). Similarly, the surface of a sample of contact-charged pyrene-TCNQ/PDMS presents the positively charged CT crystal domains and negatively charged PDMS domains with KPFM mapping. Upon illumination of both samples, the charges on both positive and negative domains decay rapidly, suggesting a rapid charge transfer between the domains. (Undoped PDMS does not show any potential change for the same time interval.) When the potential decay rates at only the crystal regions of the KPFM maps of 1-aminopyrene-TCNQ/PDMS and pyrene-TCNQ/PDMS are compared, the former CTC is faster. Before illumination, for 1-aminopyrene-TCNQ/PDMS and pyrene-TCNQ/PDMS, the charges on the crystal domains are +5.0 V and +6.0 V, respectively. After illumination for 2 min, these values drop to 0 V and +4.0 V (Figure 6, KPFM potentials of the dashed lines in the related maps). The faster discharge in the 1-aminopyrene-TCNQ regions is presumably due to the assembly’s higher degree of charge transfer and lower HOMO–LUMO gap compared to those of pyrene-TCNQ (Figure 3).

**Figure 6:**
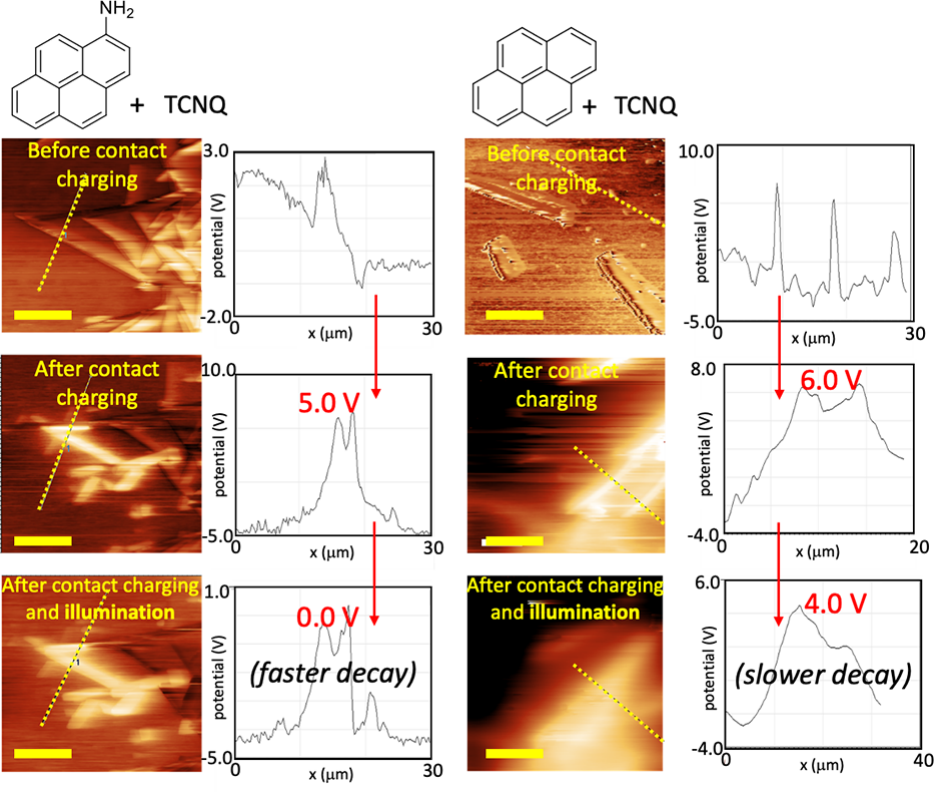
Kelvin probe force microscopy (KPFM) surface potential maps of PDMS surfaces doped with 1-aminopyrene-TCNQ (left) and pyrene-TCNQ (right) before contact, after contact charging, and charging followed by 2 min UV illumination. (The potential data are shown on the right for the dashed line crossing through the *CTC domain* in the corresponding images). The maps show CTC domain acquires positive and PDMS domains acquire negative potential. Upon illumination, the electric potential is reduced rapidly in both domains (in comparison to no decay in undoped PDMS in this time interval (not shown here). The decay rate is faster for 1-aminopyrene-TCNQ doped PDMS, where the *CTC domain* charge drops to 0 V (from +5.0 V) upon illumination. Scale bar = 10 μm.

Taking the KFM results into account, one can conclude that for successful charge dissipation, the charge should be transferred from the mechanospecies to the crystal. We can surmise this charge transfer works via a hopping mechanism on a continuous crystal with a low donor and acceptor distance (Figure S7). Such a low intermolecular distance leading to the close packing with strong interaction between donor and acceptor will provide higher charge transfer integral (T) and orbital coupling strength (H) as well as lower reorganization energy (λ) that leads to improved charge carrier mobilities according to the Marcus theory.^48^ In our case, this distance was found to be lowest in the donors with −OH and −NH_2_ substitutions on the pyrene, for which the TCNQ assemblies showed an increased charge dissipation efficiency. Other factors also affect charge mobility in charge transfer complexes, such as paracrystallinity,^49^ which is defined as a measure of cumulative deformation in molecular site positions, molecular vibrations,^50^ and long-range molecular ordering. In this study, we refrain from discussions of these other aspects due to the weak definition of microscale crystalline behavior^51^ from the nanoscale structure determined by theoretical studies.

Finally, we also probed the fast discharging ability of our CTCs for the dissipation of charges in polymers charged by corona charging instead of contact charging. For this test, we charged the 1-aminopyrene/TCNQ-PDMS (1.0 × 10^–2^ M) and undoped pieces with a negative corona discharge from the Zerostat instrument (Sigma-Aldrich) held 10 cm away from the sample upon charging. When the charge accumulated on the pieces reached about 0.5 nC, we recorded the decay of charges with time, immersing the pieces in the Faraday cup connected to the electrometer. The discharge profiles show no significant change between the decay rate in the doped and undoped pieces in dark or under UV (Figure S8). This result is presumably because the charges formed on the polymers by corona charging are the deposited ions; there is no bond breakage to form polymer mechanoions. The adhered ions do not interact with the CTCs on the surfaces.

## Conclusion

Polymers can get contact charged, since their surfaces are prone to mechanical damage and bond breaking even at the slightest contact (hence the name “contact charging”). In the research for antistatic materials, efforts can be made to dissipate the formed charges without altering the -useful- insulating nature of the polymers. It was shown before that contact-charge dissipation can be facilitated by electron transfer through low-energy pathways provided by metallocene or organometallic additives.^52^ However, these methods include high-cost materials in high additive concentrations in the polymers. With this report, we have shown that organic CTCs can be used as antistatic additives for polymers. The charge dissipation can further be tuned by the strength of the D–A charge transfer interaction and can further be facilitated (remotely) by light. The dissipation mechanisms introduced in this study can be used to understand the contact-charge dissipation mechanisms in polymers, and design more efficient, low-cost, metal-free antistatic polymeric materials.

**Figure 7:**
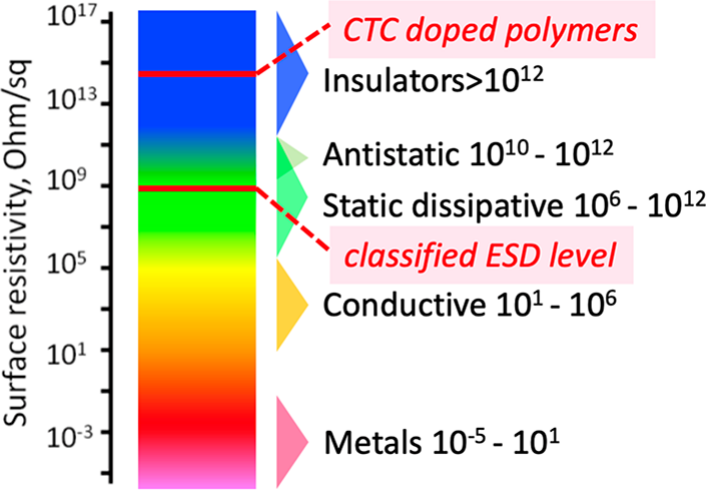
Controlling static electricity without increasing surface conductivity. In the existing approaches, materials such as ionic conductors, carbon or metal-filled resins, or conducting polymers are used to increase the conductivity of the materials to a minimum ESD classified level. However, it is possible to obtain antistatic materials without altering the surface conductivity/resistivity of the insulator polymers, e.g., with CTC-doped polymers shown in this study. See the Experimental Section for the description of ESD, surface resistivity, and its measurement.

We feel it is necessary to include a final note here on the insulating nature of the polymers retained after doping. The formed composites are “antistatic” and “insulator”. In the recent years, we^28,31^ and others^29^ have shown some examples of this behavior, which was not considered before in the field (Figure 7). Therefore, we suggest that the definition of the “antistatic” nature of any material should be considered free of the electrical resistivity of the material.

## Experimental Section

All solvents purchased from Sigma-Aldrich were used without further purification. Pyrene derivatives and TCNQ (Sigma-Aldrich) were used as obtained. PDMS was prepared by using a Dow Corning Sylgard 184 silicone elastomer kit. The samples for the electrical potential measurements using the tapping device described in the text were mounted on aluminum stubs from Agar Scientific.

We prepared a prepolymer mix of PDMS (10:1, prepolymer: base) and mixed the resulting mixture vigorously until homogenization. After removing air bubbles in a vacuum, we cured the mixture on PS Petri dishes for 24 h at 70 °C. PDMS samples (discs with a radius of 0.9 cm and a thickness of 1.52 cm were cut and washed with dichloromethane for 24 h. Once these pieces are dried overnight, they are immersed in vials of a 1:1 molar ratio of the selected donor–acceptor pair solution in dichloromethane (30 mL, each compound 1.0 × 10^–2^ M).

The absorption spectra were recorded using a Cary 5000 UV–visible spectrophotometer from Agilent. For AFM/KFM imaging, a Nanosurf AFM microscope was used.

### Tapping Setup and *V*_oc_ Measurements

The charging behavior of CTC-doped and undoped PDMS were monitored using a method that utilizes a homemade tapping device attached to an oscilloscope. A two-channel oscilloscope was used to independently measure the open circuit electrical potential (in volts) generated during the contact and separation of the two surfaces. Polymer samples were mounted on aluminum stubs connected to a 100 mega ohm (input impedance) oscilloscope probe, and an Al metal stub was attached to an identical second probe. The open-circuit voltages (*V*_oc_) were measured and collected from saturated signals (signals obtained when accumulated charges are at their maximum values). A 5 Hz tapping frequency was used. Standard deviations were calculated from at least four independent measurements. Unless otherwise stated, RH is 23–28%.

### Charge Density and Open-Circuit Potential Measurements

.CTC-doped and undoped PDMS samples were contact charged against aluminum foil for up to 10 touches. Electrostatic charges on the polymer surfaces were measured by immersing the polymer pieces in a homemade Faraday cup attached to an electrometer (Keithley 6517B).

### Charge Decay Measurements

Before charge decay experiments, PDMS pieces were left to discharge in an isolated container for at least 24 h. The electroneutrality of these pieces was confirmed by immersing the pieces in a homemade Faraday cup connected to a high-precision electrometer (Keithley Instruments, model 6517B) that measures electrical charge. Undoped and doped PDMS pieces were charged against aluminum foil several times to reach the highest surface charge (charge saturation point). Then, samples were immersed in the homemade Faraday cup for up to 30 min. Charge decay rates were calculated using Excel by linear equation fitting.

### Surface Resistivity/Conductivity Measurements

The electrical resistance of the surface of insulator material is termed its surface resistivity. The values of surface resistivities are measured from electrode to electrode along the surface of the insulator sample. According to ASTM D-257, DC Resistance or Conductance of Insulating Materials, surface resistivity is determined from the measurement of surface resistance between two electrodes forming opposite sides of a square. Since the surface length is fixed, the measurement is independent of the physical dimensions of the insulator sample. The standard spectrum shown in Figure 7 is obtained by comparing charge dissipation rates and the surface resistivities of the surfaces in general. The safe ESD threshold is termed as “classified ESD level”.

Surface resistivities of pyrene, 1-aminopyrene, TCNQ, CTC1, and CTC2 doped and undoped PDMS were measured using a two-probe method, with *w* = 17 mm wide samples, and the distance between electrodes *d* = 217 μm. The values of surface conductivity, κ, are calculated according to equation κ = 1/ρ, p= *R*(*w*/*d*). A Keithley electrometer (6517B) also served as the voltage source using a two-wire resistance measurement setting, where 100 V was applied for four samples in each group, giving identical *R* values. The surface conductivities (thickness independent) are shown in Table S2.

### Computational Methods

The HOMO and the LUMO energy levels for pristine TCNQ acceptor and pyrene donor with different substitutions were calculated for the optimized geometries using DFT methods with the M06-2X functional^53^ and the 6-311G(d) basis set with tight convergence criteria in Gaussian09.^54^ The structures of the individual molecules at the anionic and cationic states were also optimized at the same calculation level to calculate the adiabatic ionization potentials (IP) and electron affinities (EA) using the neutral, anionic, and cationic geometry energies. The geometries of the binary systems formed by the acceptor and each donor molecule were optimized, and D–A charge transfers based on the electrostatic potential fitting (ESP)^55^ and natural population analysis (NPA)^56^ were calculated. The HOMO and LUMO of the binary interaction of D–A charge transfer complex systems were mapped onto the optimized structures. The same process was applied for the quaternary charge transfer complexes; in addition to the HOMO and LUMO, HOMO–1 and LUMO+1 were mapped onto the optimized quaternary structures. Counterpoise corrected interaction energies between donor and acceptor were calculated for the lowest energy structures of binary and quaternary complexes. Charge transfer was also calculated by the method proposed by Kistenmacher et al.^57^ by using the bond lengths of the optimized individual acceptor molecule and the optimized acceptor molecule in interaction with the donor functionalized with different substituents.

Frontier orbitals were also calculated at the excited state optimized geometry to elucidate the effect of photoexcitation on the electronic structure of these charge transfer cocrystals. Natural transition orbitals from S_0_ → S_1_ levels for both ground state and excited state optimized geometries were determined by the TDDFT method by calculating the first 40 excited states and their oscillatory frequencies.^58−60^

## Supporting Information Available

Solution and solid-state UV–visible spectra, calculated charge decay rates, XRD diffractograms of CTC in PDMS, measured surface conductivities of PDMS samples, the details about the HOMO–LUMO and charge transfer value calculations (PDF)
Movie S1, discharging of PTFE beads in a solution of 1-aminopyrene-TCNQ (MP4)
Movie S2, discharging of PTFE beads in a solution of 1-aminopyrene (MP4)
