# Supplementary material for: Organic Charge Transfer Cocrystals as Additives for Dissipation of Contact Charges on Polymers
Source: ACS Appl Mater Interfaces. 2022 Dec 6;14(50):56018–26. doi: 10.1021/acsami.2c13643 (PMC9782351; doi:10.1021/acsami.2c13643)
Supplement: Supplementary file 1 — am2c13643_si_001.pdf [file am2c13643_si_001.pdf]

Supporting Information

Organic Charge Transfer Cocrystals as Additives for Dissipation of Contact Charges on Polymers

Sunay Dilara Ekim[1], Görkem Eylül Kaya[1], Murat Daştemir[2], Erol Yıldırım[2,3]*, H. Tarik Baytekin[2,3]*, and Bilge Baytekin[1,4]*

[1] UNAM National Nanotechnology Research Center, Bilkent University, 06800 Ankara, Turkey
[2] Department of Chemistry, Middle East Technical University, 06800 Ankara, Turkey
[3] Polymer Science and Technology Program, Middle East Technical University, 06800 Ankara, Turkey
[4] Department of Chemistry, Bilkent University, 06800 Ankara, Turkey

*Email: erolyil@metu.edu.tr
*Email: tarikbay@metu.edu.tr
*Email: b-baytekin@fen.bilkent.edu.tr

Supporting Movies

Movie S1. CT assembly mediated discharging of contact charged polymer beads in hexane. a) PTFE beads in 8.0 mL hexane are contact charged by shaking on a vortexer for 30 sec. The charged beads "stick" electrostatically to the walls of the vials. Then a solution of 1-aminopyrene-TCNQ (acceptor) (1:1, final concentration 3.12×10^{-6} M, dry hexane) is added to glass vials. The beads discharge in ca. 500 seconds and fall to the bottom of the vial. UV illumination speeds up the process.

Movie S2. Pyrene and its derivatives alone cannot discharge contact-charged polymer beads in hexane. a) PTFE beads in 8.0 mL hexane are contact charged by shaking on a vortexer for 30 sec. The charged beads "stick" electrostatically to the walls of the vials. Then a solution of 1-aminopyrene (final concentration 3.12×10^{-6} M, dry hexane) is added to glass vials. The beads do not discharge in 500 seconds. UV illumination does not help the discharge, either.

Supporting Experimental Results and Figures

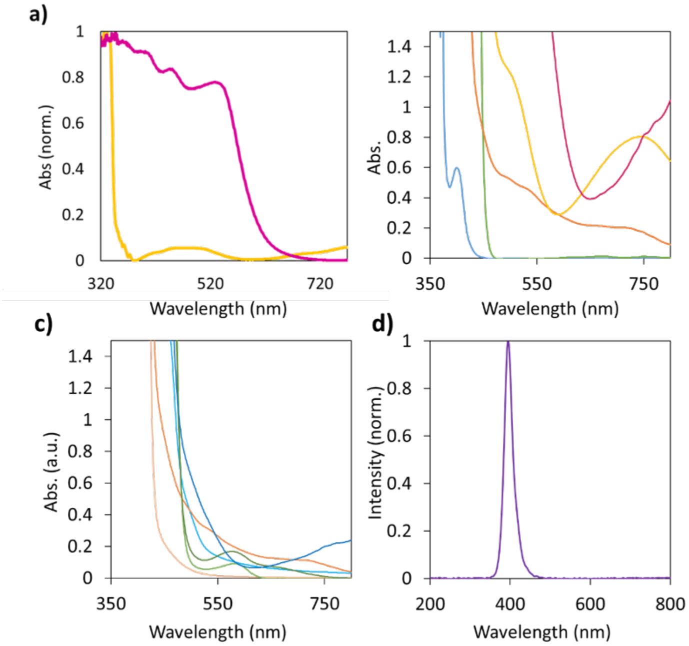

Figure S1. a) Solid-state UV-visible spectra of pyrene-TCNQ (yellow) and 1-Aminopyrene-TCNQ (pink). The charge transfer bands are the broad peaks in pyrene-TCNQ (488 nm and 777 nm) and 1-aminopyrene-TCNQ doped PDMS (450- 625 nm). (b) UV-visible spectra of CTCs with their acceptor and donors in dichloromethane (1.0 x 10^{-2} M) - pyrene-TCNQ (yellow), 1-Aminopyrene-TCNQ (pink), 1-Aminopyrene (orange), TCNQ (green), Pyrene (blue). (c) UV-visible spectra of other donors and CTCs in the study, in dichloromethane (1.0 x 10^{-2} M): 1-pyrenecarboxaldehyde (light orange), 1-pyrenecarboxaldehyde -TCNQ (dark orange), 1-nitropyrene (light green), 1-nitropyrene/TCNQ (dark green), 1-Hydroxypyrene (light blue), and 1-Hydroxypyrene/TCNQ (dark blue). The distinctive red-shifted broad bands for CTCs are the charge transfer bands that do not exist in the spectra of the donors. d) The emission profile of UV led source used for illuminating samples in the solid state and in-solution charge decay experiments.

Table S1. The decay rate of contact charges on the CTC-doped PDMS samples. The charge decay profiles for the first 30 mins of decay, shown in Figure 4, are fitted to first-order kinetics to obtain the rate constants. *= The decay is fitted to second-order kinetics. Please see Experimental for the details of preparation of the samples, the charging and decay experiments.

	**Mean ± SD**
**Sample**	**Rate Constants (s^{-1})**
PDMS Dark	0.002 ± 0.001
PDMS UV	0.002 ± 0.001
Pyrene Dark	0.002 ± 0.000
Pyrene UV	0.002 ± 0.001
1-Aminopyrene Dark	0.006 ± 0.002
1-Aminopyrene UV	0.024 ± 0.008
TCNQ Dark	0.061 ± 0.022
TCNQ UV	0.063 ± 0.006
Pyrene-TCNQ Dark	0.006 ± 0.001
Pyrene-TCNQ UV	0.014 ± 0.006
1-Aminopyrene-TCNQ Dark*	0.349 ± 0.143
1-Aminopyrene-TCNQ UV*	0.115 ± 0.028

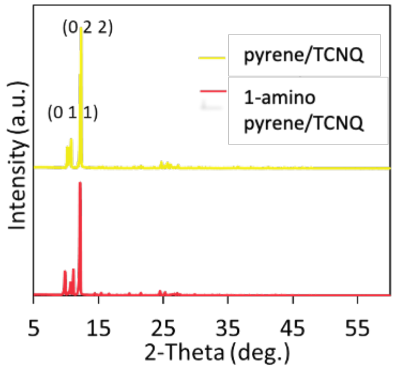

Figure S2. X-ray diffractogram of pyrene-TCNQ doped PDMS (yellow) and 1-Aminopyrene-TCNQ doped PDMS (red). The PDMS samples were doped from $1x10^{-2}$ M CTC solutions.

Table S2. Surface conductivities of CTC-doped PDMS. Please see Experimental for the details of preparation of the samples, and the surface conductivity experiments.

Sample	Conductivity (S)
PDMS Dark	9.45×10^{-14}
PDMS UV	9.44×10^{-14}
Pyrene Dark	8.86×10^{-14}
Pyrene UV	8.82×10^{-14}
1-Aminopyrene Dark	8.33×10^{-14}
1-Aminopyrene UV	8.33×10^{-14}
TCNQ Dark	7.82×10^{-14}
TCNQ UV	7.67×10^{-14}
Pyrene-TCNQ Dark	5.39×10^{-14}
Pyrene-TCNQ UV	5.37×10^{-14}
1-Aminopyrene-TCNQ Dark*	5.55×10^{-14}
1-Aminopyrene-TCNQ UV*	5.55×10^{-14}

Supporting Computational Results and Figures

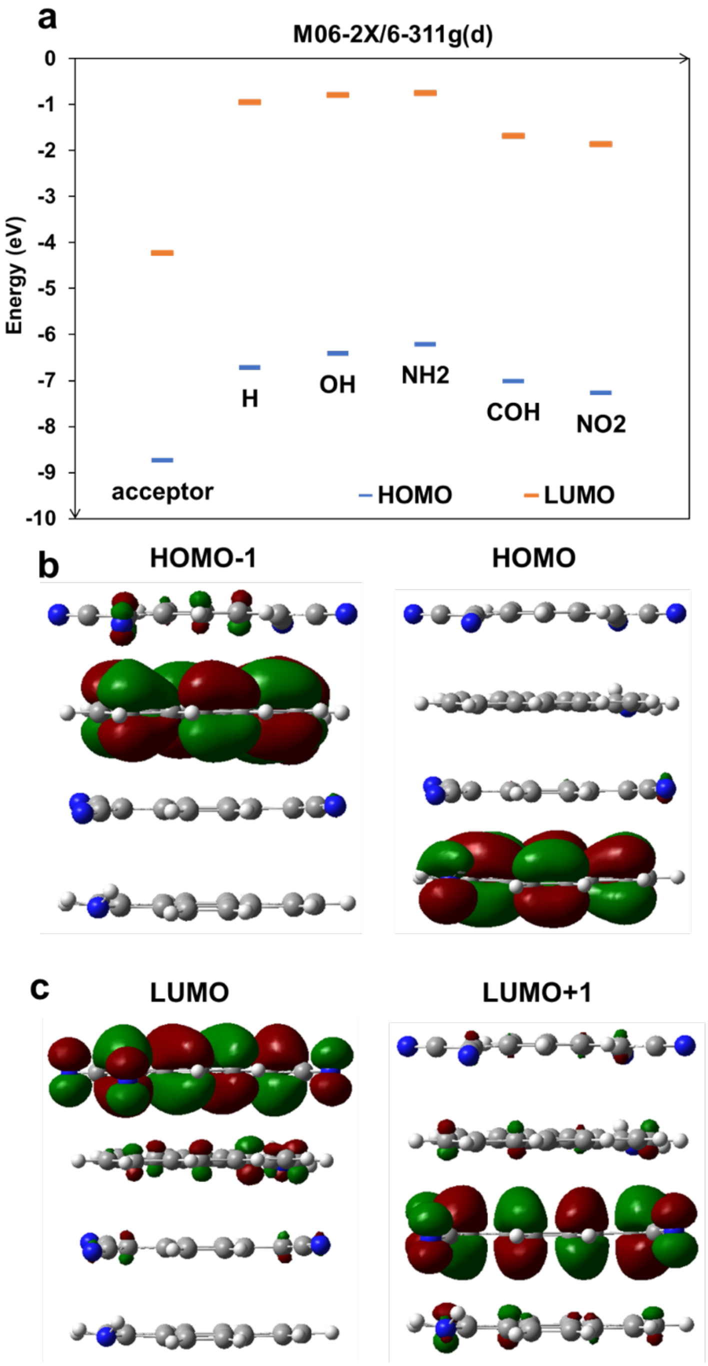

Figure S3. a) HOMO and LUMO energy levels for the acceptor and the donor with different substitutions, b) HOMO and HOMO-1 orbitals, c) LUMO and LUMO+1 orbitals mapped on the quadruple interactions of donor-acceptor charge transfer complex for -NH_2 substitutions on pyrene donor.

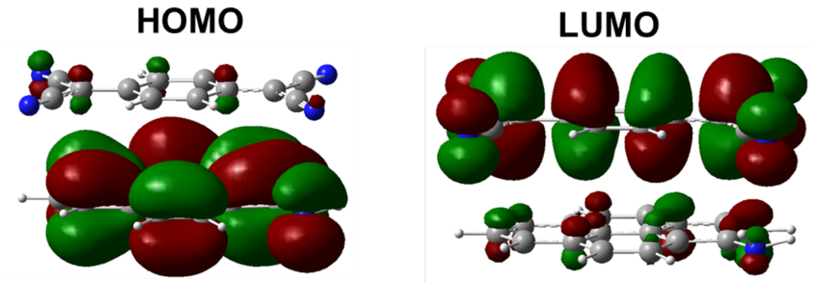

Figure S4. HOMO and LUMO orbitals mapped on donor-acceptor charge transfer complex pairwise interactions for $-NH_2$ substitutions on pyrene donor.

To help understand the charge transfer mechanism between the acceptor and substituted donor derivatives, the geometries of the pristine single molecule structures (TCNQ, pyrene, and pyrene derivatives) were optimized, and ground state energy levels were determined at M06-2X/6-311g(d) level of calculations (Figure S3). Various pairwise complex geometries were constructed by varying intermolecular distance, and their respective orientation was subjected to geometry optimization to determine the lowest energy structures for the binary complex. According to the results obtained, it was concluded that the minimum energy pairwise geometries are formed by the π-stacking of the donor and acceptor molecules, as expected (Figure S4). This π-stacked structure model was used to construct initial guesses for quadruple charge transfer complex geometries. For all binary and quaternary complexes, The HOMO and LUMO of the pairwise CTCs structures clearly demonstrated that the HOMOs of all the pairwise CTCs are on the donor molecules and the LUMOs are on the acceptor molecule (Fig. S3-S4). HOMO-1 orbitals are on the donor, and LUMO+1 orbitals are on the acceptor for the quaternary complex (Figure S3b). Two donor molecules in quadruple CTCs bear HOMO and HOMO-1 on them, while the two acceptor molecules bear LUMO and LUMO+1. Similar trends for frontier orbital energy levels and their distributions were observed using B3LYP-D3 and wB97XD functionals. When the HOMO and LUMO energy levels of the optimized pristine donor and acceptor structures are examined (Figure S3a), it can be clearly observed that as the difference between the HOMO energy level of the donor molecule and the LUMO energy level of the acceptor molecule decreased, the charge transfer between the donor-acceptor complex. The dissipation of contact charges was enhanced in the discharge experiments in this order, too (Figure 3, main text). According to all DFT calculations with different functionalities, the LUMO of the acceptor has the closest value to the HOMO of the pyrene donors with $-NH_2$ and -OH substitution. These donors show better performance for charge dissipation than the others.

Intermolecular charge transfer was observed by all atomic charge calculation methods. The charge transfer values for pairwise and quadruple CTCs obtained by the ground state electrostatic potential (ESP) and natural population analysis (NPA) are listed in Table S3. Both charge calculation algorithms predict the same trends. As can be seen in Table S3, TCNQ/1-aminopyrene and TCNQ/1-hydroxypyrene have greater charge transfer values compared to the other CTCs. For the different pyrene substitutions, as the difference between donor-HOMO and acceptor-LUMO energy levels decreases, charge transfer increases. The adiabatic ionization potential values for the donor molecules (Table S3-S4) were obtained by taking the difference between the energy of the optimized positively charged molecule and the energy of the optimized neutral molecule. For the acceptor molecule, the adiabatic electron affinity (Table S4) was calculated by taking the difference between the energy of the optimized negatively charged TCNQ and the energy of the optimized neutral TCNQ. For enhanced charge transfer, it was already known that the HOMO energy of the donor should be close to the LUMO energy of the acceptor and that the donor should have a lower ionization potential[56]. Considering Table S3, the enhancement of charge transfer in CTCs can be achieved using a donor molecule with a lower ionization potential. We found that the smaller the IP of the donor, the more effective the charge transfer as calculated lowest for with amine and hydroxy substitutions of pyrene donor. Pyrenes with these substitutions also have the least negative EA values. Considering the HOMO energy levels and ionization potentials among the donor molecules, 1-aminopyrene and 1-hydroxypyrene have the best values as donor molecules in the CTCs used in the experiments.

The interaction energies for pairwise and quadruple structures are given in Table S3, which indicates that the interaction energies between donor and acceptor in TCNQQ/1-aminopyrene and TCNQ/1-hydroxypyrene complexes are greater than in other CTCs, also decreases the intermolecular distances with acceptor for these two donors.

Table S3. Effect of donor substitution on the charge transfers, IP and EA differences, and interaction energies between the donor (D) and acceptor (A) for pairwise and quadruple interactions.

**CT complexes**	**Pairwise interactions**				**Quadruple Interactions**		
**Donor substitution**	**ESP**	**NPA**	**Interaction Energy (kcal/mole)**	**IP−|EA| (eV)**	**ESP**	**NPA**	**Interaction Energy (kcal/mole)**
**$-NH_2$**	-0.184	-0.107	-24.63	3.002	-0.847	-0.26	-70.70
**-OH**	-0.142	-0.079	-24.77	3.372	-0.829	-0.19	-69.53
**-H**	-0.135	-0.070	-21.76	3.736	-0.788	-0.17	-63.39
**-COH**	-0.116	-0.063	-21.54	3.997	-0.497	-0.15	-62.90
**$-NO_2$**	-0.096	-0.059	-21.05	4.256	-0.445	-0.16	-60.12

Table S4. EA values were calculated at the M062x/6-311g(d) level by taking the energy difference between the optimized anion and neutral structures. IP values were calculated at the M062x/6-311g(d) level by taking the energy difference of the optimized cation and neutral structures. Calculations are for the acceptor and donor separately.

	**Electron Affinity (eV)**	**Ionization Potential (eV)**
Acceptor	-3.68	9.38
Donor($-NH_2$)	-0.14	6.68
Donor-(OH)	-0.15	7.05
Donor(-H)	-0.29	7.41
Donor(-COH)	-1.11	7.67
Donor($-NO_2$)	-1.45	7.93

Another factor that governs the charge transfer is the $IP_{(donor)} - |EA|_{(acceptor)}$ offset value. A linear relationship between the magnitude of charge transfer and IP − |EA| was demonstrated (Figure S5a). We determined that the smaller the IP of the donor, the more effective the doping is in increasing the electrical conductivity. These results agree with experimental results and explain the molecular level mechanism for the dissipation of contact charges. Adiabatic ionization potentials of donor molecules and the adiabatic electron affinity of the acceptor molecule were used to obtain IP − |EA| offset. In situations where IP − |EA| offset is small, the CTCs are more ionic in character, allowing more charge transfer. Zhu et al. [56] showed that charge transfer vs. IP − |EA| plot falls onto a straight line for various CTCs containing 2,3,5,6 tetrafluoro-7,7,8,8-tetracyanoquinodimethane (F4-TCNQ). Similarly, when the NPA charge transfer for pairwise CTCs (Table S3) and IP − |EA| (Table S3) is plotted, a linear relationship is observed, as given in Figure S5a.

The bond length change in the TCNQ molecule can also be utilized to predict the amount of charge transfer. To calculate the amount of charge transfer, ρ, from the bond length changes of TCNQ, we use the method proposed by Kistenmacher et al. [53].

$$\boldsymbol{\rho} = -\frac{\alpha_{CT}-\alpha_0}{\alpha_{-1}-\alpha_0} \quad \text{with } \boldsymbol{\alpha_i} = \frac{\ell_1}{\ell_2+\ell_3}$$

Where ℓ is the bond length described in Figure S6 and subscript i = 0, −1, CT: denotes the neutral molecule, the anion, and the complex, respectively. According to Table S5, it can be concluded that the interaction between TCNQ with donor molecules causes the lengthening of the double bond ℓ_1 and the shortening of two single bonds, ℓ_2 and ℓ_3, all three of which stand for the most notable changes. Our results were validated by the α_0 value for TCNQ given as 0.476 experimentally[54,55] and calculated as 0.475 at M06-2X and 0.482 at B3LYP-D3/6-311g(d) level. When the charge transfer calculated by the bond length changes, $CT_{\text{bond length}}\,\rho$, versus the charge transfer obtained by the NPA charges is plotted (see Figure S5b), a relationship emerges where amine substituted pyrene should show the most improved charge dissipation performance - which is experimentally observed.

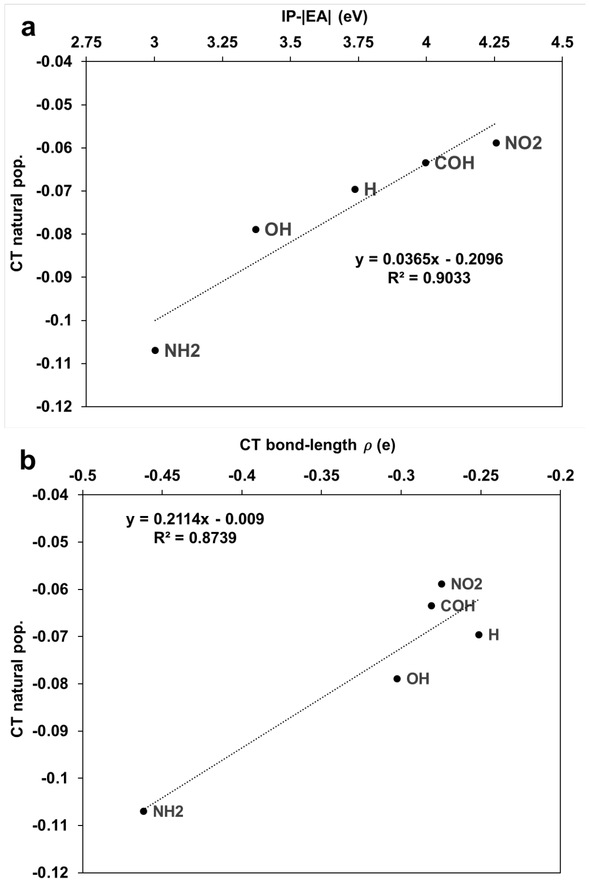

Figure S5. a) Correlation of IP−|EA| offset with the charge transfer based on NPA. b) Correlation of charge transfer from the bond lengths of the acceptor (ρ) with the charge transfer based on the NPA.

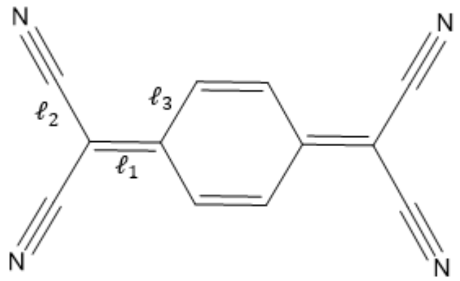

Figure S6. The bonds in the TCNQ acceptor used for calculating charge transfer.

Table S5. The bond lengths in the TCNQ acceptor obtained for the optimized geometry of neutral, anion, and in pairwise interaction with the donor.

**Structure**	**ℓ_1 (Å)**	**ℓ_2 (Å)**	**ℓ_3 (Å)**
Acceptor (anion)	1.42877	1.41162	1.4241
Acceptor (neutral)	1.38492	1.42342	1.44636
Acceptor- Donor(-NH_2)	1.39664	1.42061	1.4363
Acceptor- Donor(-OH)	1.38792	1.42184	1.44323
Acceptor- Donor(-H)	1.38509	1.42278	1.44491
Acceptor- Donor(-COH)	1.38694	1.42245	1.44412
Acceptor- Donor(-NO_2)	1.38616	1.42191	1.4441

At last, we calculated the excited state properties of the binary complexes to elucidate the effect of photoexcitation via illumination. In the excited state, the intermolecular distance between the donor and acceptor molecules was decreased. This decrease in the intermolecular distance is especially more significant for hydroxy and amine substitution (Fig. S6a). Moreover, the most probable natural transition orbitals for the ground state to the first singlet excited state based on the highest oscillatory frequency and occupation were determined for both ground state and excited state geometries, where increased electronic transfer and coupling between molecules were observed via excitation by the structural change due to photoexcitation as shown for amine substitution (Figure S6b-c). The main finding of the excited state calculations via illumination is on the band gap, for which the values calculated for the ground state binary complex geometry decreased dramatically for hydroxy, followed by amine substitutions in the excited state geometry due to the decreased intermolecular distances and electronic rearrangement in the excited state. One can expect enhanced dissipation of contact charges on polymers via illumination for the hydroxy and amine substituted pyrene donor-based CTCs due to these decreased intermolecular distance, lowered band gap, and enhanced coupling with acceptor via photoexcitation. (Fig. S6d)

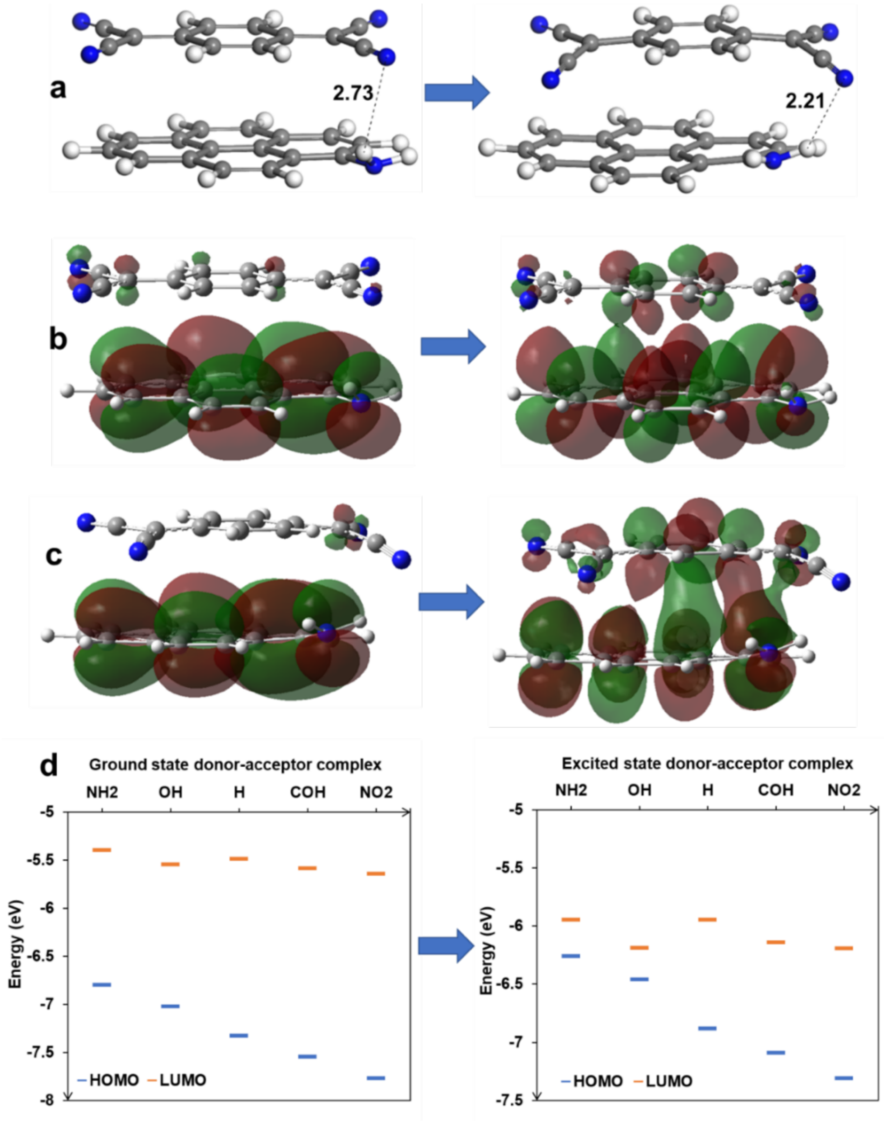

Figure S6. a) Optimized geometries for the ground state and first singlet excited state pairwise interactions for -NH_2 substitution, $S_0 \rightarrow S_1$ natural transition orbitals at the b) ground state optimized geometries, and c) excited state optimized geometries, d) HOMO and LUMO energy levels for the ground state and excited state optimized geometries of binary complexes for different substitutions on the pyrene donor.

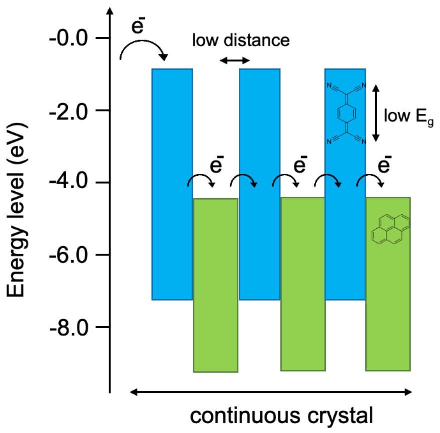

Figure S7. The surmised hopping mechanism for charge dissipation. Electrons from polymer mechanospecies are transferred to crystal. The charge is then carried in the crystal – most efficiently if the physical separation between the donor-acceptor is low.

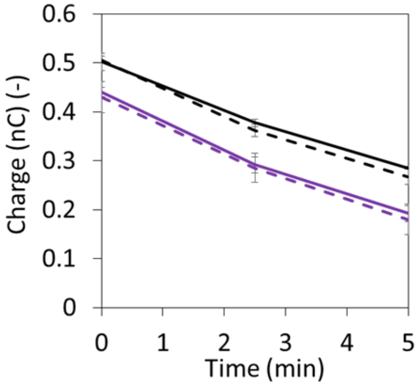

Figure S8. 1-aminopyrene-TCNQ doped and undoped PDMS pieces are corona-charged with a negative corona discharge from the Zerostat instrument (Sigma-Aldrich) held 10 cm from the sample upon charging. The charges are left to decay in a Faraday cup connected to an electrometer recording charges. The recorded charge decay for doped (dashed lines) and undoped (solid lines) pieces have similar rates both in the dark (black lines) and under UV illumination (purple lines). Error bars correspond to standard deviations determined from at least four independent experiments. CT assembly is doped into PDMS by immersing the pieces in 1.0 x 10^{-2} M 1:1 assembly solution in dichloromethane. See Experimental for further details on sample preparation, and discharge measurement setup.
